# Evaluating and presenting uncertainty in model‐based unconstrained ordination

**DOI:** 10.1002/ece3.5752

**Published:** 2019-12-20

**Authors:** Andrew Hoegh, David W. Roberts

**Affiliations:** ^1^ Department of Mathematical Sciences Montana State University Bozeman MT USA; ^2^ Department of Ecology Montana State University Bozeman MT USA

**Keywords:** Bayesian estimation, latent factor models, multivariate species models, ordination

## Abstract

Variability in ecological community composition is often analyzed by recording the presence or abundance of taxa in sample units, calculating a symmetric matrix of pairwise distances or dissimilarities among sample units and then mapping the resulting matrix to a low‐dimensional representation through methods collectively called ordination. Unconstrained ordination only uses taxon composition data, without any environmental or experimental covariates, to infer latent compositional gradients associated with the sampling units. Commonly, such distance‐based methods have been used for ordination, but recently there has been a shift toward model‐based approaches. Model‐based unconstrained ordinations are commonly formulated using a Bayesian latent factor model that permits uncertainty assessment for parameters, including the latent factors that correspond to gradients in community composition. While model‐based methods have the additional benefit of addressing uncertainty in the estimated gradients, typically the current practice is to report point estimates without summarizing uncertainty. To demonstrate the uncertainty present in model‐based unconstrained ordination, the well‐known spider and dune data sets were analyzed and shown to have large uncertainty in the ordination projections. Hence to understand the factors that contribute to the uncertainty, simulation studies were conducted to assess the impact of additional sampling units or species to help inform future ordination studies that seek to minimize variability in the latent factors. Accurate reporting of uncertainty is an important part of transparency in the scientific process; thus, a model‐based approach that accounts for uncertainty is valuable. An R package, UncertainOrd, contains visualization tools that accurately represent estimates of the gradients in community composition in the presence of uncertainty.

## Graphical abstract

Variability in ecological community composition is often visualized with a low‐dimensional ordination projection. However, most existing approaches ignore the uncertainty present in this process. This manuscript details methods for understanding uncertainty in unconstrained ordination.

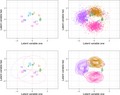

## 1 INTRODUCTION

Multivariate ordination, and in particular model‐based ordination with latent factor modeling, is used by community ecologists to understand patterns in community composition. Data on species presence/absence or abundance are used in ordination methods to identify compositional similarities between sample units. With these ordination models, the goal is not necessarily to learn about the individual species themselves, but rather the underlying gradients that influence species distributions and ultimately community composition. However, many data sets do not contain sufficient information to discern precise estimates of the ecological gradients that influence community composition.

In contrast to constrained ordination methods where environmental features related to the sample units are used (Anderson & Willis, 2003; Birks, Peglar, & Austin, 1996; Økland, 1996), unconstrained ordination models do not include ancillary data about the sample units, but rather only use species (or other taxon) composition information to estimate the locations of sample units along compositional gradients. Traditionally, distance‐based methods have been used to determine the compositional gradients and locations of sample units (Legendre & Gallagher, 2001; Roberts, 2016); however, these methods require resampling‐based approaches for inference and uncertainty assessment (De Leeuw & Meulman, 1986; Heiser & Meulman, 1983; Jacoby & Armstrong, 2014; Smith & Gray, 2019). Recently, model‐based methods have been introduced for unconstrained ordination (Hui, Taskinen, Pledger, Foster, & Warton, 2015; Ovaskainen et al., 2017; Warton, Blanchet, et al., 2015; Warton, Foster, De'ath, Stoklosa, & Dunstan, 2015), which do permit uncertainty assessment. A common implementation of model‐based unconstrained ordination uses a latent factor model (Hui et al., 2015) where the latent gradient of community composition is estimated as an unobserved latent variable.

In Hui et al. (2015), four benefits of model‐based approaches to unconstrained ordination are detailed as follows: controlling spurious data properties, model checking, model selection and inference, and efficiency; however, little attention is given to assessing uncertainty in model parameters. In fact, the only mention of confidence intervals in this section states that “accuracy of such confidence intervals in this context is in need of evaluation.” A later paper with a Bayesian implementation (Hui, 2016) largely resolves the issue of accuracy of the intervals and while other recent articles (Hui, Tanaka, & Warton, 2018; Hui, Warton, Ormerod, Haapaniemi, & Taskinen, 2017; Niku, Warton, Hui, & Taskinen, 2017) do touch on variability, understanding and assessing uncertainty in the latent factors is still not a point of emphasis. Walker (2015) does include an analysis of uncertainty in indirect gradient analysis, but the scope is limited to a single dimensional projection of presence/absence data. Ren, Bacallado, Favaro, Holmes, and Trippa (2017) details an approach using a dependent Dirichlet process that implements for understanding uncertainty in projections for bacteria counts, but is limited to count data. Understanding and evaluation of uncertainty is critical, in science in general and particularly in unconstrained ordination, as estimating latent gradients with precision from abundance or presence data is a major challenge. With unconstrained ordination, parameter estimation is challenging because, in addition to the latent factors, species exhibit significantly varying patterns of occurrence or abundance which also need to be estimated. Furthermore, many ecological data sets are sparse in that many species are rare, and the number of observations can be small compared to the number of parameters required to be estimated. The focus of this work is to assess uncertainty in latent factors; quantify the effects of the number of sample units or species and the relative width of uncertainty bounds; and encourage accurate reporting of the variability; additionally, tools are given for designing ordination studies in a way that reduces the variability in the final results.

Bayesian methods have become quite popular for both latent factor models (Lopes & West, 2004) and in ecological analyses modeling joint species data (Gelfand et al., 2005; Halstead, Wylie, Coates, Valcarcel, & Casazza, 2012; Ovaskainen et al., 2017; Pollock et al., 2014; Taylor‐Rodriguez, Kaufeld, Schliep, Clark, & Gelfand, 2017; Walker, 2015). A major benefit of the Bayesian modeling framework is that uncertainty in model parameters can be assessed with the posterior distribution, typically expressed by calculating credible intervals.

In many statistical situations, increased precision in parameter estimates is attained by collecting additional data. In this framework, the latent factors are associated with the sample units, and hence, increased precision would be attained by collecting abundance or presence data for additional species at each sampling location. While there are sampling protocols in some ecological disciplines where a fixed number of organisms is identified for each sample unit, in many cases sampling is exhaustive and the number of species per sample unit cannot be increased. Furthermore, the inclusion of rare species results in sparse data where some species are rarely observed. The sparse data do not provide much information about the latent factors associated with the sample units. Fortunately, increasing the number of sample units does have an indirect impact on the latent factors associated with an community composition gradient as it allows the species parameters to be more precisely estimated, which, in turn, results in more precise site‐level estimation. The trade‐offs between collecting additional species or data at additional sites will be addressed in Section 4.

To determine the characteristics required to achieve a specified precision in estimating the underlying gradients, we present a framework for addressing the sample unit characteristics necessary to detect the latent factors with a specified precision. Precision is impacted by the number of sample units, the number of species, and the assumed generating distribution of the data, but also by the factors associated with the abundance distribution for each species and each sample unit. Simulation studies are presented for a variety of scenarios, and code is made available with our R package, UncertainOrd, that is available on github (Hoegh, 2018).

In this article, we detail the challenges in using model‐based methods for unconstrained ordination and emphasize the importance of understanding and relaying the uncertainty in estimates. Furthermore, in addition to providing tools to calculate the characteristics required to detect underlying gradients at a specified precision level, we also provide software for creating figures that display the uncertainty in the parameters.

## 2 MATERIALS AND METHODS

There is a variety of ways to collect data for species response. Common responses include presence/absence, counts of individuals, and percent cover which is typically reported using ordinal cover classes. Presence/absence and count responses are self‐explanatory. Percent cover is often reported in discrete ordinal categories, such as Daubenmire's cover classes (Daubenmire, 1959), which, when including zero, has six classes: 0, 0%–5%, 5%–25%, 25%–50%, 50%–75%, 75%–95%, and 95%–100%. Regardless of the response type, statistical distributions and model‐based approaches are available for each scenario.

A common approach for model‐based unconstrained ordination uses latent factor models. Following notation from Hui et al. (2015), the latent factor model with random effects for sample units can be formulated as

(1) $$g\left(\mu_{\mathrm{ij}}\right)=\alpha_{i}+\beta_{j}+z_{i}^{T}\theta_{j},$$

where *α_i_* represents the effect for site *i*, *β_j_* is the effect for species *j*, *z_i_* = (*z_i_*_1_, …, *z_iq_*) is the *q*‐dimensional vector of latent factors, at site *i*, each of which is assumed to be independent and standard normal in distribution, and *θ_j_* are the factor loadings associated with the latent factors. The function *g*() is a generic function used to link the mean term *μ_ij_* to the assumed generative distribution of the data. The *z_i_*'s are referred to as latent factors and are assumed to represent the underlying gradient that influences community composition. Typically, *q*, the dimension of the latent factors, is two or three for ordination. Without constraints on the vector of *θ_j_* values, the latent variables are not invariant to rotation. Hence with *q* = 2, for identifiability ***θ*** is constrained such that *θ*_11_ > 0, *θ*_12_ = 0, and *θ*_21_ > 0, where *θ_jk_* is the coefficient, or factor loading, associated with the *j*th species and *k*th dimension of the latent factor.

All variables in Equation (1) are fit using a Bayesian paradigm. Hence, the following model specifications and prior distributions are used

(2) $$\alpha_{i}∼N\left(\mu_{\alpha },V_{\alpha }\right)$$

(3) $$\beta_{j}∼N\left(\mu_{\beta },V_{\beta }\right)$$

(4) $$\theta_{j}∼N\left(0,V_{\theta }\right)$$

(5) $$z_{i}∼N\left(0,I\right),$$

where *μ_α_* and *μ_β_* are the mean parameters for the prior distributions for *α* and *β*, respectively, and are usually set to zero. Variance in the prior distributions is specified as *V_α_*, *V_β_*, or *V_θ_*, which can be a covariance matrix. To satisfy the constraints on *θ*, the upper diagonal element, *θ*_12_, is set to zero and the diagonal elements *θ*_11_ and *θ*_22_ are forced to be positive by using a truncated normal distribution as the prior. The latent factors are assumed to follow a standard normal distribution.

Unconstrained ordination approaches using only species abundance, presence/absence, or ordinal responses tend to have large uncertainty in the estimates of the latent factors; however, understanding the impact of the number of species and number of sample units on uncertainty in the latent factors is an important part of planning an ordination study. Regardless of the sampling model of the data, a posterior distribution for the latent factors can be examined to assess the uncertainty in the projections. In practice, there are a collection of ways to summarize the posterior distribution. We present tools to visualize the uncertainty in the ordination projection of the latent factors. The uncertainty can be presented as a collection of points a 95% highest posterior density (HPD) interval, or density representation of the posterior distribution. Simulation studies are created to understand how the number of species and the number of sampling units impact the uncertainty in the posterior distribution of the latent factors.

### 2.1 Models for binary data

Multivariate species data are often recorded in a presence/absence format. This results in binary data, and there are two common approaches for this data structure: logistic regression using the logit link and probit regression using a probit link. Generally, in a Bayesian framework, which we adopt in this article, the probit model specification is preferred due to efficient sampling. In particular, a Gibbs sampler can be implemented with the probit link function using a latent normal data augmentation approach (Albert & Chib, 1993; Chib & Greenberg, 1998). However, the recent advances in the Polya‐Gamma random variables and efficient sampling also permit Gibbs sampling in the logistic case (Polson, Scott, & Windle, 2013). In this article, we will focus on the probit regression, but the results are quite similar using logistic regression.

The probit regression uses a data augmentation approach to facilitate efficient computation of the posterior distribution. In this case, we assume there is a latent, continuous variable such that if the latent variable is <0, then the observed binary response is equal to zero; otherwise, if the latent variable is >0, then the observed binary response is equal to one. Then, the model can be written as

(6) $$y_{\mathrm{ij}}∼\text{Bernoulli}\left(\mu_{\mathrm{ij}}\right)$$

(7) $$\mu_{\mathrm{ij}}=\Phi \left(\alpha_{i}+\beta_{j}+z_{i}^{T}\theta_{j}\right)$$

where *y_ij_* is the binary response for species *i* at location *j* and Φ() is the cumulative distribution function of a standard normal random variable. The prior distributions from Equations ((2), (3), (4)) are used for parameter estimation.

### 2.2 Models for count data

Multivariate species data are often observed as an abundance, where the recorded value is the count of the number of individuals by species. Count data are commonly modeled with the Poisson distribution; however, the Poisson distribution has a strict mean/variance property that is not always realistic. Negative binomial models provide an alternative that has a more flexible structure to fit data where the mean and variance are not equal as in a Poisson model.

#### 2.2.1 Poisson model

The Poisson model can be written as

(8) $$y_{\mathrm{ij}}∼\text{Poisson}\left(\mu_{\mathrm{ij}}\right)$$

(9) $$\mu_{\mathrm{ij}}=\exp\left(\alpha_{i}+\beta_{j}+z_{i}^{T}\theta_{j}\right)$$

where *y_ij_* is the count response for species *i* at location *j*. The prior distributions from Equations ((2), (3), (4)) are used for parameter estimation.

#### 2.2.2 Negative binomial distribution

The negative binomial is quite similar to the Poisson distribution, but also includes an overdispersion parameter to account for extra variation in the counts. Specifically, the model can be written as

(10) $$y_{\mathrm{ij}}∼\text{Negative}\;\text{Binomial}\;\left(\text{mean}=\mu_{\mathrm{ij}},\;\text{overdispersion}=\omega \right)$$

(11) $$\mu_{\mathrm{ij}}=\exp\left(\alpha_{i}+\beta_{j}+z_{i}^{T}\theta_{j}\right),$$

note that this parameterization of the negative binomial distribution is different from many approaches that use the number of trials and a probability of success (or failure). With this parameterization, the variance of $y_{\mathrm{ij}}=\mu_{\mathrm{ij}}+\omega \mu_{\mathrm{ij}}^{2}$. This framework also requires a prior distribution on the overdispersion parameter *ω*. Common prior distributions for *ω* include a half‐normal distribution or a half‐Cauchy distribution.

### 2.3 Ordinal data

In some cases rather than counts or presence, the response of interest is the percent cover of each species. In this case, rather than estimating a percent, values are often recorded in ordinal responses. When modeling ordinal data, a common approach uses the cumulative probit link function. Building off the probit model from Equation (7), and extending to *k* categories, the goal is estimating the probabilities of the outcome being in each of the *k* classes, $\left\{\mathrm{Pr}\left[Y_{\mathrm{ij}}=1\right],\ldots ,\mathrm{Pr}\left[Y_{\mathrm{ij}}=k\right]\right\}$. To estimate these probabilities, a latent variable is constructed as

(12) $$z_{\mathrm{ij}}^{*}∼N\left(\alpha_{i}+\beta_{j}+z_{i}^{T}\theta_{j},1\right),$$

where

(13) $$y_{\mathrm{ij}}=\left\{\begin{array}{c} 1\;\text{if}\;z_{\mathrm{ij}}^{*}<c_{1}\\ 2\;\text{if}\;c_{1}\leq z_{\mathrm{ij}}^{*}<c_{2}\\ \vdots \\ k-1\;\text{if}\;c_{k-1}\leq z_{\mathrm{ij}}^{*}<c_{k}\\ k\;\text{if}\;z_{\mathrm{ij}}^{*}>c_{k} \end{array}\right.$$

where *c_l_* is the *l*th cutoff point. The variance is set to be one for identifiability in this framework. Then for a given species and site combination, the cumulative probit model is used to determine the probability of each class.

## 3 RESULTS

In this section, we analyze two well‐known data sets to illustrate the uncertainty inherent in model‐based unconstrained ordination.

### 3.1 Spider data set

The spider data set was first published in Van der Aart and Smeenk‐Enserink (1974) and contains counts for 12 species of spiders at 28 sampling sites. The spider data set is generally considered information dense as more than 54% of the values are >0 with an average total abundance across all species and sites of nearly ten. In model‐based unconstrained ordination, the focus is the latent factors which are then used as coordinates in a low‐dimensional projection.

#### 3.1.1 Spider presence analysis

We begin with treating the spider data set as a binary response by mapping the abundance data to presence data. Naturally, treating the data as binary provides less information for identifying the latent factors. Nevertheless, many data sets are collected as presence/absence data and this provides a direct measure of the effect of information loss when compared to the spider abundance data below.

Using the probit link function, we fit the model specified in Equations (6 and 7) using a two‐dimensional latent variable *Z* = {*z*_1_, *z*_2_}. Priors were specified from the distributions in Equations ((2), (3), (4)) where the hyperparameters were set as $\mu_{\alpha }=\mu_{\beta }=0$, $\sigma_{\alpha }^{2}=\sigma_{\beta }^{2}=1$, and $V_{\theta }=I$. Our interest is the latent factors *z*_1_ and *z*_2_ which are used to interpret a latent gradient associated with the 28 sample units. Using a Bayesian framework, uncertainty in the latent factors can be assessed with the posterior distribution.

There is a large amount of uncertainty in all of the latent factors; in fact, the posterior distribution contains zero for each variable. The interest of the study is not in a traditional hypothesis test to determine whether the variables are different from zero, but nevertheless proper accounting of uncertainty is important in the scientific process.

**Figure 1:**
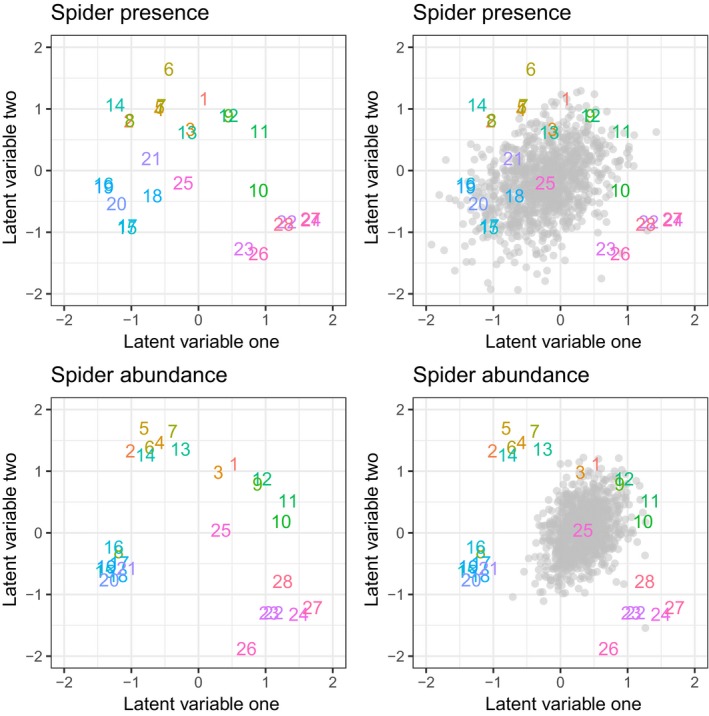
Point estimates of latent factors for the spider data are shown in the left two panels, where the top row contains information from the spider presence analysis and the bottom row is from the spider abundance analysis. The right panels also contain the posterior distribution of site 25 as the point cloud. The uncertainty is smaller for the abundance data set than in the presence data analysis, but the variability is still relatively large

To illustrate the uncertainty in the latent factors, the posterior distribution of a single site, number 25, was plotted in the top right corner of Figure 1. Site 25 was chosen because it was near the center of the figure and most of the posterior distribution would fit on the plot with the same scale as the left part of the figure which only contains the point estimates of the latent factors. The interpretation is that the posterior mean would be centered at the number 25 on the figure, but the value could be at any of the gray points in the background. This uncertainty should encourage caution in reporting a single‐point estimate for the projection of latent factors, like those on the left panels of Figure 1.

#### 3.1.2 Spider abundance analysis

The spider data set was also analyzed using the recorded counts of individuals by species. The abundance data set contains more information than the presence data set, as it better represents variability in species response to the latent factors. While the Poisson distribution could be used here, Hui et al. (2015) found the negative binomial to be a better fit to the spider data. Additionally, deviance information criteria (DIC; Spiegelhalter, Best, Carlin, & Linde, 2002) are used to compare the two models and we also find a negative binomial to be more appropriate for this data. Hence, the negative binomial model specified in Equation (10) is used in this example. With this specification, standard normal priors are placed on *α*, *β*, *Z*, and *θ*. The dispersion term in the negative binomial distribution has a half‐Cauchy prior with variance of 20.

Similar to the abundance data analysis, the uncertainty in the latent factors can be visualized. The parameters exhibit less uncertainty than do the parameters for the presence/absence data, but there is still a large amount of variability present. Figure 1 shows the point estimates of the latent factors using the negative binomial sampling model along with the variability for site 25.

### 3.2 Dune data set

The dune data set is another famous data set that contains the abundances of 30 plant species collected across 20 sampling sites. The data set was reported in Batterink and Wijffels (1983) in Dutch and later in Jongman, Braak, and Tongeren (1995). The response is collected using a Braun‐Blanquet method with nine ordinal classes. Roughly, two‐thirds of the responses are zero, which denotes no presence of the species. Of the 30 species, 10 are recoded in three sampling units or fewer; more specifically, three of the species are only recorded in one plot, two species are recorded in two plots, and five species are recorded in three plots. The most abundant species, in terms of presence across a number of plots, occurs in 18 of the plots.

#### 3.2.1 Dune ordinal data analysis

The ordinal data analysis uses standard normal priors for *z*, *θ*, *α*, and *β*. The ordinal model specified in Equations (12 and 13) also requires a prior distribution for the cutoffs. A standard normal prior is used here too with the constraint that order of the cutoffs is appropriately preserved.

**Figure 2:**
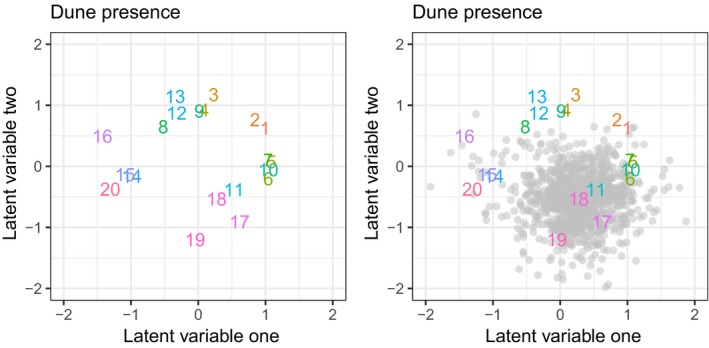
Point estimates of latent factors for the dune data are shown in the left panel. The right panel also contains the posterior distribution of site 18 as the point cloud

The data are analyzed, and a point estimate of latent factors is presented in the left panel of Figure 2. Additionally, the uncertainty for site 18 is presented in the right panel of Figure 2.

### 3.3 Data analysis summary

The figures of the dune data set and the spider data set suggest there is a high level of uncertainty in the projections from model‐based ordinations; however, they do not provide an easy way to quantify the uncertainty. Before describing ways to quantify and compare uncertainty, we first note that with these ordination approaches, the interest is more in relative differences than absolute differences. In other words, the interest is not in the absolute value of the latent factors used for projection, but rather in the relative differences between the positions for sampling locations.

Relative differences can be visualized by anchoring one of the sampling sites at (0,0) on the projection figures. Then, the posterior samples for the other sites are transformed as distances from the anchored point. This controls for cases where the entire set of ordination values shift together.

**Figure 3:**
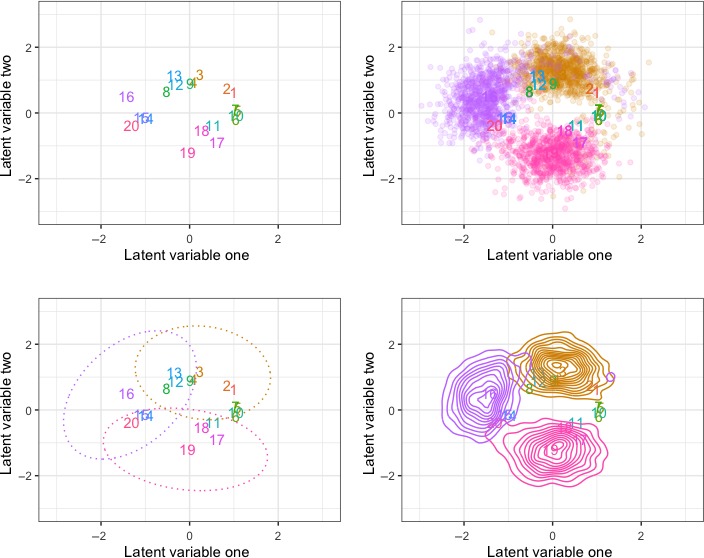
Point estimates of latent factors for the dune data are shown in the top left panel. The other three figures contain various ways of displaying the uncertainty in the projections, where the colors of the ellipses or point clouds correspond to similarly colored numbers

Figure 3 shows four ways to visualize the relative uncertainties of the projected latent factors for the dune analysis. The top left shows the mean of the relative differences from site 18. In essence, the figure is shifted so that site 18 is anchored at (0,0). The three other figures show combinations of 95% highest posterior densities of the latent factors and points of posterior samples for selected sites. Again, these are relative locations or distances to site 18.

Another way to summarize the uncertainty in the estimated latent factors is to look at the width of the posterior credible intervals. In particular, we considered the 95% HPD posterior intervals for the latent factors and report the average width of these intervals. The average width of the HPD intervals was 2.51 and 1.73 for presence and abundance, respectively, for the spider data set and 2.15 for the dune data set. The one‐dimensional gradient used in Walker (2015) for the dune data set had similar levels of uncertainty. With the spider data set, abundance data models have substantially smaller levels of uncertainty than presence data. Hence to limit uncertainty, whenever possible, abundance data should be collected rather than absence or presence.

There are a few differences between the dune and spider data sets that explain the differences in HPD interval widths. To further explore the differences, the dune data set was converted to presence/absence and analyzed using the same priors as the spider analysis, resulting in a mean HPD width of 2.01. In comparing the average HPD width for the spider and dune data sets, in a binary setting, the dune data set has a narrower posterior interval. This is due to a combination of factors. The dune data set has a larger number of species, which likely leads to the increased precision in the latent factors; however, the dune data contains more zeros (67% of species/site combinations are zero) compared to the spider data set (45% of species/site combinations are zero). Comparing the results for the abundance data, the count data appear to contain more information than the ordinal percent cover data. The impact of the number of species observed as well as species rarity and data set sparsity will be further addressed in Section 4.

Unfortunately, ecologists have more control over the number of sample units than the number of species that occur in a sample unit. In addition, including species that very rarely occur does not provide much information for estimating the latent factors. Fortunately, obtaining more sample units is helpful in that the species parameters are more precisely estimated which results in more precise estimates of site parameters as well.

Finally, it is not always possible to achieve a small amount of uncertainty in the latent factors; hence, we strongly urge reporting results in a way that reflects the uncertainty. In our R package, UncertainOrd, MCMC samples can be extracted to give two‐dimensional HPD credible intervals around each latent factor. Using the dune analysis, Figure 3 contains examples of ways to show the projection of the latent factors that respect the uncertainty.

## 4 SIMULATION STUDY AND EFFECT SIZE ESTIMATION

This section presents a set of simulation studies designed to assess the uncertainty in the latent factors as a function of the number of species, species frequency of occurrence, and the number of sites. To simulate species responses, the framework in Equation (1) is used. The species and site effects are randomly sampled from normal distributions with mean *μ_α_* and *μ_β_* and standard deviations *σ_α_* and *σ_β_*, respectively. These values are explicitly detailed for each separate simulation study. For all simulation studies, *θ* and *z* are simulated from standard normal distributions. Given the species and site effects as well as *θ* and *z*, raw species counts are simulated. The simulations are set up using a factorial design for both the number of species and the number of sample units.

### 4.1 Presence/absence versus abundance simulations

To evaluate uncertainty in presence/absence and abundance ordinations, credible interval widths for latent factor estimates were assessed with a simulation analysis varying the number of species and sites. The goal is to understand the impact of *n* and *p* on credible interval width of the latent factors and compare across presence/absence and abundance data. Synthetic data were generated from a Poisson distribution with $\mu_{\alpha }=\mu_{\beta }=0$ and $\sigma_{\alpha }=\sigma_{\beta }=1$. Note that Section 4.3 describes an alternative scenario where these values can be estimated from a pilot study and specified. A factorial structure was used with 10, 25, 50, 75, and 100 units for species and sampling sites. Fifty replicates were included for each pair of species and sampling sites. The data were then analyzed as both a count response and a binary response, assuming only presence/absence is recorded.

**Figure 4:**
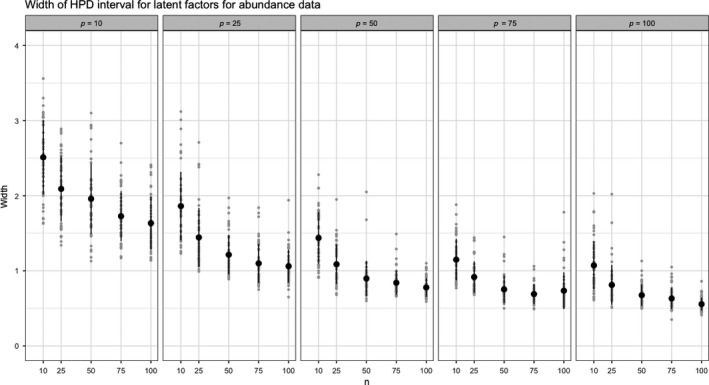
Average HPD width for abundance data with specified *n* and *p* based on 50 replicates

**Figure 5:**
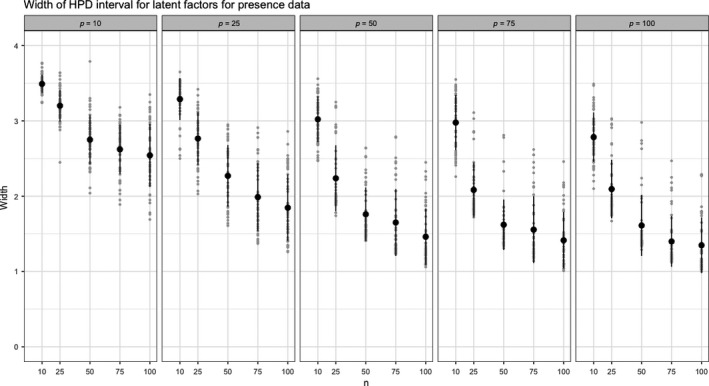
Average HPD width for presence data with specified *n* and *p* based on 50 replicates

The uncertainty in the latent factors for both scenarios can be seen in Figures 4 and 5. The abundance data allow more precise estimation of the latent factors. This is not surprising as useful information is discarded when counts are mapped to presence/absence. Thus, whenever possible, counts rather than presence should be recorded.

### 4.2 Sparsity simulation

In ordination studies, utility of the species data is to learn about the sites. Accordingly, observing more species is one option for increasing the information content of the data. Unfortunately, these additional species may rarely occur; hence, we investigate the impact of adding rare species on the uncertainty in the estimates of the latent factors. To explore the effect of infrequently occurring species, we created a simulation using presence/absence data where rare species are included. Note, common heuristics suggest dropping species that occur less than *m* times, where *m* is generally 5 or less.

For all simulations, the number of sites was fixed at 20. This value for the number of sites is arbitrary, but puts the focus on impacts from changing the species data. We considered two scenarios: first a standard presence/absence data structure where the species effects are simulated from a standard normal distribution and secondly a framework where a subset of the species are extremely rare. For both cases, standard normal distributions are used for simulating *Z* and *θ*. The site effects, *α*, are simulated from a normal distribution with mean of zero and standard deviation of 0.5 for all of the standard species. In general, this results in a hypothetical species being observed 50% of the time or at 10 sites, on average. Then, we consider a data set that is augmented with rare species that have effects simulated from a normal distribution with mean of −4 and standard deviation of 0.5. Approximately 75% of the simulated rare species had zero occurrences across the 20 sites. Across all combinations of site and species, the probability of occurrence is 0.027.

The width of the HPD intervals for the latent factors, calculated as the average width of the latent factors across the replicates, can be seen in Table 1.

**Table 1:** Average width of the HPD intervals for the latent factors based on 50 replicates

*p*	Width	*p*_rare_	Width
10	2.77	5 + 5	2.83
20	2.18	10 + 10	2.30
40	1.74	20 + 20	1.83
80	1.12	40 + 40	1.24

Unsurprisingly, the data that include the rarer species has more uncertainty in the width of the credible intervals. However, note that the rare species do include some additional information such that 10 species plus 10 rare species contains more information than just 10 regular species. Furthermore, the information from 10 commonly occurring species and 10 rare species does lead to more uncertainty than 20 commonly occurring species.

### 4.3 Interval width estimation

To better assist researchers designing ordination studies, we created a function, (HPD width) in the R package UncertainOrd, that allows users to enter various parameters and evaluate the anticipated HPD width both numerically and visually. The function takes the following arguments: family of the sampling distribution, *n*, *p*, *μ_α_*, *μ_β_*, *σ_α_*, and *σ_β_*.

When designing a study, a common question is how many sites/species should be estimated. This simulation looks at the trade‐off between *n* and *p* with parameters that control abundance and presence that relate to a specific data set. As an example, we revisited the spider data set to examine the impacts of changing *n* and *p* on the expected HPD width of the latent factors. Using the observed parameter values of the spiders data, we simulated data sets with additional species or sample units. The estimated parameters for all of these data sets are available in Table 2.

**Table 2:** Estimated parameters for *α* and *β* for spider data set

	*μ_α_*	*SD_α_*	*μ_β_*	*SD_β_*
Spider presence	0.04	0.73	0.10	1.02
Spider abundance	0.03	0.66	0.05	1.08

Note

These values represent the average across all species or sample units.

**Figure 6:**
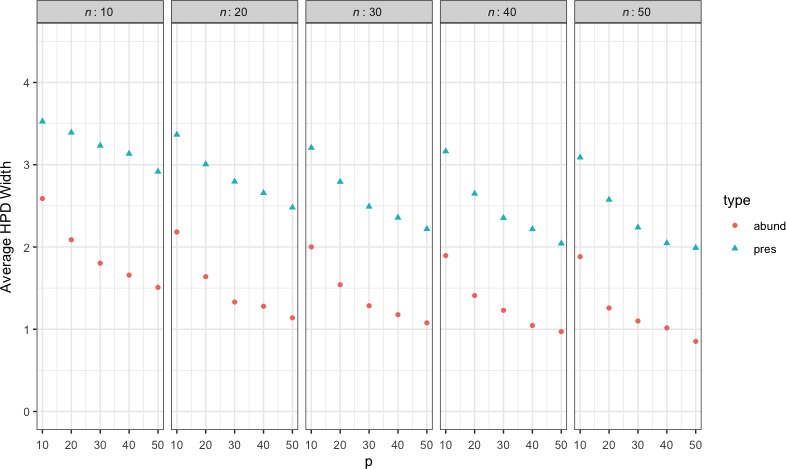
Average HPD credible interval width for simulated scenarios using population characteristics from spider data set. With *n* = 28 and *p* = 12 the average width of the HPD intervals was 1.72 and 2.95 for abundance and presence respectively

Figure 6 presents the population characteristics of the spider data set used to estimate HPD width of the latent factors for a variety of values of *n* and *p*. In this experiment, a factorial structure was used with 10, 20, 30, 40, and 50 observations for the number of species and the number of sites. Fifty replicates were run for each combination of species and sites.

In general, the results suggest that increasing the species count is slightly preferable to increasing the number of sites when the goal is to minimize the width of the credible intervals. For instance, with 40 sites and 50 species the HPD interval width of the latent factors is 0.97 and 2.04 which is smaller than 1.05 and 2.22 for 50 sites and 40 species. Given constraints on an experiment, this tool will allows researchers to use data‐driven methods to choose the appropriate number of species or sites.

## 5 DISCUSSION

The goal of this research was to examine the uncertainty inherent in model‐based unconstrained ordination and to provide tools for accurate reporting of the uncertainty in the latent factors associated with community composition gradients. The tools allow researchers to plan for uncertainty due to controllable factors, such as the number of sample units and species to observe. We strongly recommend that model‐based ordination results include some representation of uncertainty.

Model‐based methods, using Bayesian credible intervals, provide a way to estimate uncertainty in model parameters associated with latent gradients. Large variability in gradients exhibited by these methods is not a reason to eschew model‐based methods; rather on the contrary, accounting for uncertainty is an essential element in the scientific process.

In general, larger numbers of species and sampling locations help limit uncertainty in the underlying gradients. While observing a large number of total units at a site influences the uncertainty in the latent factors, the total number of sites also plays a role. Where collecting species composition at additional sites also can help reduce variability in the latent factors. Collecting data as an abundance rather than presence/absence is recommended as this also helps limit uncertainty.

Given the complexity of latent factor models in solving for community composition gradients and the lack of additional information outside of species presence/absence or abundance, uncertainty is an unavoidable part of unconstrained ordination. Hence, we have created tools that permit projections in the estimates of the gradients that account for and express the uncertainty present. Accounting for and reporting uncertainty is an essential part of the scientific process and being transparent in the knowledge gained from a scientific study. These tools presented here can be used by ecologists to in the same manner as standard ordination projections, such as determining overlap between sampling units based on the species composition; however, credible regions account for uncertainty and provide valid statistical inferences.

## CONFLICT OF INTEREST

None declared.

## AUTHOR CONTRIBUTIONS

AH and DWR jointly identified research idea. AH implemented methods/software, conducted data analysis, and wrote manuscript. AH and DWR edited and approved manuscript.

## Data Availability

All data used in the manuscript are already publicly accessible. The dune data set is available in the vegan package in R (Oksanen et al., 2018). The spider data set is available in the mvabund package in R (Wang, Naumann, Eddelbuettel, & Warton, 2018).

## References

[ece35752-bib-0001] Albert, J. H. , & Chib, S. (1993). Bayesian analysis of binary and polychotomous response data. Journal of the American Statistical Association, 88(422), 669–679. 10.1080/01621459.1993.10476321

[ece35752-bib-0002] Anderson, M. J. , & Willis, T. J. (2003). Canonical analysis of principal coordinates: A useful method of constrained ordination for ecology. Ecology, 84(2), 511–525. 10.1890/0012-9658(2003)084[0511:CAOPCA]2.0.CO;2

[ece35752-bib-0003] Batterink, M. , & Wijffels, G. (1983). Een vergelijkend vegetatiekundig onderzoek naar de typologie en invloeden van beheer van 1973 tot 1982 in de duinweilanden op Terschelling, Landbouwhogeschool.

[ece35752-bib-0004] Birks, H. J. B. , Peglar, S. M. , & Austin, H. A. (1996). An annotated bibliography of canonical correspondence analysis and related constrained ordination methods 1986–1993. Abstracta Botanica, 20, 17–36.

[ece35752-bib-0005] Chib, S. , & Greenberg, E. (1998). Analysis of multivariate probit models. Biometrika, 85(2), 347–361. 10.1093/biomet/85.2.347

[ece35752-bib-0006] Daubenmire, R. (1959). Plants and environment. Hoboken, NJ: Wiley.

[ece35752-bib-0007] De Leeuw, J. , & Meulman, J. (1986). A special jackknife for multidimensional scaling. Journal of Classification, 3(1), 97–112. 10.1007/BF01896814

[ece35752-bib-0008] Gelfand, A. E. , Schmidt, A. M. , Wu, S. , Silander, J. A. , Latimer, A. , & Rebelo, A. G. (2005). Modelling species diversity through species level hierarchical modelling. Journal of the Royal Statistical Society: Series C (Applied Statistics), 54(1), 1–20. 10.1111/j.1467-9876.2005.00466.x

[ece35752-bib-0009] Halstead, B. , Wylie, G. , Coates, P. , Valcarcel, P. , & Casazza, M. (2012). ‘Exciting statistics’: The rapid development and promising future of hierarchical models for population ecology. Animal Conservation, 15(2), 133–135.

[ece35752-bib-0010] Heiser, W. J. , & Meulman, J. (1983). Constrained multidimensional scaling, including confirmation. Applied Psychological Measurement, 7(4), 381–404.

[ece35752-bib-0011] Hoegh, A. (2018). UncertainOrd: An R package for Unconstrained Ordination. https://github.com/andyhoegh/UncertainOrd

[ece35752-bib-0012] Hui, F. K. (2016). Boral‐Bayesian ordination and regression analysis of multivariate abundance data in R. Methods in Ecology and Evolution, 7(6), 744–750.

[ece35752-bib-0013] Hui, F. K. , Tanaka, E. , & Warton, D. I. (2018). Order selection and sparsity in latent variable models via the ordered factor lasso. Biometrics, 74(4), 1311–1319. 10.1111/biom.12888 29750847

[ece35752-bib-0014] Hui, F. K. , Taskinen, S. , Pledger, S. , Foster, S. D. , & Warton, D. I. (2015). Model‐based approaches to unconstrained ordination. Methods in Ecology and Evolution, 6(4), 399–411. 10.1111/2041-210X.12236

[ece35752-bib-0015] Hui, F. K. C. , Warton, D. I. , Ormerod, J. T. , Haapaniemi, V. , & Taskinen, S. (2017). Variational approximations for generalized linear latent variable models. Journal of Computational and Graphical Statistics, 26(1), 35–43. 10.1080/10618600.2016.1164708

[ece35752-bib-0016] Jacoby, W. G. , & Armstrong, D. A. (2014). Bootstrap confidence regions for multidimensional scaling solutions. American Journal of Political Science, 58(1), 264–278.

[ece35752-bib-0017] Jongman, R. , Ter Braak, C. , & Van Tongeren, O. (1995). Data analysis in community and landscape ecology. Cambridge, UK: Cambridge University Press.

[ece35752-bib-0018] Legendre, P. , & Gallagher, E. D. (2001). Ecologically meaningful transformations for ordination of species data. Oecologia, 129(2), 271–280. 10.1007/s004420100716 28547606

[ece35752-bib-0019] Lopes, H. F. , & West, M. (2004). Bayesian model assessment in factor analysis. Statistica Sinica, 14, 41–67.

[ece35752-bib-0020] Niku, J. , Warton, D. I. , Hui, F. K. , & Taskinen, S. (2017). Generalized linear latent variable models for multivariate count and biomass data in ecology. Journal of Agricultural Biological and Environmental Statistics, 22(4), 498–522. 10.1007/s13253-017-0304-7

[ece35752-bib-0021] Økland, R. H. (1996). Are ordination and constrained ordination alternative or complementary strategies in general ecological studies? Journal of Vegetation Science, 7(2), 289–292. 10.2307/3236330

[ece35752-bib-0022] Oksanen, J. , Blanchet, F. G. , Friendly, M. , Kindt, R. , Legendre, P. , McGlinn, D. , … Wagner, H. (2018). vegan: Community ecology package. R package version 2.5‐3. Retrieved from https://CRAN.R-project.org/package=vegan

[ece35752-bib-0023] Ovaskainen, O. , Tikhonov, G. , Norberg, A. , Guillaume Blanchet, F. , Duan, L. , Dunson, D. , … Abrego, N. (2017). How to make more out of community data? A conceptual framework and its implementation as models and software. Ecology Letters, 20(5), 561–576. 10.1111/ele.12757 28317296

[ece35752-bib-0024] Pollock, L. J. , Tingley, R. , Morris, W. K. , Golding, N. , O'Hara, R. B. , Parris, K. M. , … McCarthy, M. A. (2014). Understanding co‐occurrence by modelling species simultaneously with a joint species distribution model (JSDM). Methods in Ecology and Evolution, 5(5), 397–406. 10.1111/2041-210X.12180

[ece35752-bib-0025] Polson, N. G. , Scott, J. G. , & Windle, J. (2013). Bayesian inference for logistic models using Pólya‐gamma latent variables. Journal of the American Statistical Association, 108(504), 1339–1349.

[ece35752-bib-0026] Ren, B. , Bacallado, S. , Favaro, S. , Holmes, S. , & Trippa, L. (2017). Bayesian nonparametric ordination for the analysis of microbial communities. Journal of the American Statistical Association, 112(520), 1430–1442. 10.1080/01621459.2017.1288631 29430070 PMC5804367

[ece35752-bib-0027] Roberts, D. W. (2016). labdsv: Ordination and multivariate analysis for ecology. R package version 1.8‐0.

[ece35752-bib-0028] Smith, R. J. , & Gray, A. N. (2019). Combining potentially incompatible community datasets when harmonizing forest inventories in subarctic Alaska, USA. Journal of Vegetation Science, 30(1), 18–29. 10.1111/jvs.12694

[ece35752-bib-0029] Spiegelhalter, D. J. , Best, N. G. , Carlin, B. P. , & Van Der Linde, A. (2002). Bayesian measures of model complexity and t. Journal of the Royal Statistical Society: Series B (Statistical Methodology), 64(4), 583–639.

[ece35752-bib-0030] Taylor‐Rodriguez, D. , Kaufeld, K. , Schliep, E. M. , Clark, J. S. , & Gelfand, A. E. (2017). Joint species distribution modeling: Dimension reduction using Dirichlet processes. Bayesian Analysis, 12(4), 939–967. 10.1214/16-BA1031

[ece35752-bib-0031] Van der Aart, P. , & Smeenk‐Enserink, N. (1974). Correlations between distributions of hunting spiders (Lycosidae, Ctenidae) and environmental characteristics in a dune area. Netherlands Journal of Zoology, 25(1), 1–45. 10.1163/002829675X00119

[ece35752-bib-0032] Walker, S. C. (2015). Indirect gradient analysis by Markov Chain Monte Carlo. Plant Ecology, 216(5), 697–708. 10.1007/s11258-015-0467-7

[ece35752-bib-0033] Wang, Y. , Naumann, U. , Eddelbuettel, D. , & Warton, D. (2018). mvabund: Statistical methods for analysing multivariate abundance data. R package version 3.13.1. Retrieved from https://CRAN.R-project.org/package=mvabund

[ece35752-bib-0034] Warton, D. I. , Blanchet, F. G. , O'Hara, R. B. , Ovaskainen, O. , Taskinen, S. , Walker, S. C. , & Hui, F. K. (2015). So many variables: Joint modeling in community ecology. Trends in Ecology & Evolution, 30(12), 766–779. 10.1016/j.tree.2015.09.007 26519235

[ece35752-bib-0035] Warton, D. I. , Foster, S. D. , De'ath, G. , Stoklosa, J. , & Dunstan, P. K. (2015). Model‐based thinking for community ecology. Plant Ecology, 216(5), 669–682. 10.1007/s11258-014-0366-3

